# Ovarian Clear Cell Carcinoma Presenting as Non-bacterial Thrombotic Endocarditis and Systemic Embolization

**DOI:** 10.4021/wjon367e

**Published:** 2011-10-28

**Authors:** Zaher Oueida, Michael Scola

**Affiliations:** a Internal Medicine Physician, Mayo Regional Hospital, 897 W Main Street, Dover Foxcroft, Maine 04426, USA; b Hematology Oncology Associates of Northern NJ, Carol G Simon Cancer Center, 100 Madison Ave, Carol Cancer Center, Morristown, NJ 07962, USA

**Keywords:** Non-bacterial, Thrombotic endocarditis, Marantic, Ovarian clear cell cancer, Hypercoagulable, Malignancy

## Abstract

Non-bacterial thrombotic endocarditis (NBTE) is a rare manifestation of cancer-induced hypercoaguability. It most commonly occurs in association with mucin-producing adenocarcinomas and has rarely been described with ovarian clear cell carcinoma (OCCC). We report a case of NBTE with multi-organ embolic infarcts occurring in a patient with early stage clear cell ovarian cancer. A 56 years old Caucasian female presented with leg pain, and left flank discomfort. Evaluation revealed multi-organ infarction, extensive deep vein thrombosis (DVT), and the incidental presence of an asymptomatic large ovarian mass with a laboratory picture consistent with disseminated intravascular coagulation (DIC). The diagnosis of NBTE was supported by echocardiogram and multiple negative bacteriological studies. She underwent surgical extirpation of an early stage OCCC and initiation of anticoagulation. Postoperatively, the patient’s hypercoaguability promptly resolved with gradual resolution of vegetations. Subsequent recurrence of the malignancy was heralded by a return of the prothrombotic state. This case shows a rarely reported association between NBTE and OCCC. It illustrates how the clinical picture of NBTE can dominate the initial presentation of an early stage and otherwise asymptomatic malignancy. Late recognition can lead to significant morbidity and a rapidly fatal course. Recurrent thromboembolism may be the first indication of disease recurrence.

## Introduction

Non-bacterial thrombotic endocarditis (NBTE) is an uncommon hypercoaguable condition, frequently occurring in the setting of advanced malignancy. The hallmark of NBTE is friable, valvular lesions composed of platelets interwoven with fibrin strands. Embolization is common, leading to organ infarction. Clinical manifestations depend on the site and extent of infarction. Disseminated intravascular coagulation (DIC) and other manifestations of cancer hypercoaguability often accompany NBTE. The diagnosis is challenging and often suspected by abnormal findings on echocardiogram and negative blood cultures. NBTE has been reported to occur in association with multiple malignancies including papillary serous ovarian cancer. We report an unusual case of NBTE and DIC with multi-organ embolic infarcts occurring in a patient with clear cell subtype ovarian cancer.

## Case report

A 56 year-old, previously healthy, Caucasian female was referred to our hospital for evaluation of thrombocytopenia and acute myocardial, renal, and splenic infarction. A few weeks prior to presentation, she developed acute left leg pain several weeks following surgery and physical therapy sessions for an occupational knee injury. She subsequently developed nausea and vomiting, anorexia, fevers and chills, associated with a dull, non-radiating left flank and left side chest discomfort. Her family history was notable for a mother with non-small cell lung cancer.

On physical examination, she was in no acute distress, afebrile, with a heart rate of 95 BPM and normal blood pressure. Cardiac examination demonstrated a normal rhythm, with no detectable murmurs or irregular sounds. A non-tender fullness was palpated in the right lower abdomen with some mild left upper quadrant tenderness. Non-specific induration was appreciated in the right calf without edema or venous stasis changes.

**Figure 1:**
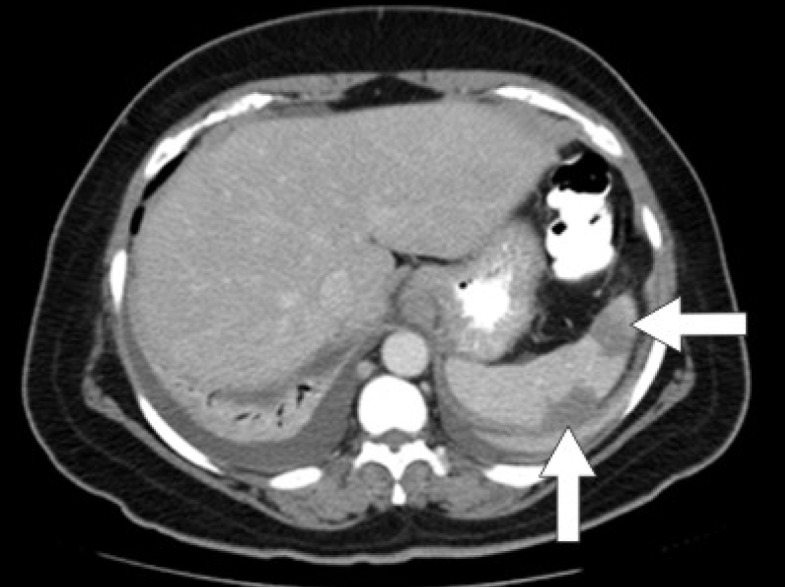
CT scan of the abdomen showing large splenic wedge-shaped hypodensities consistent with splenic infarcts.

**Figure 2:**
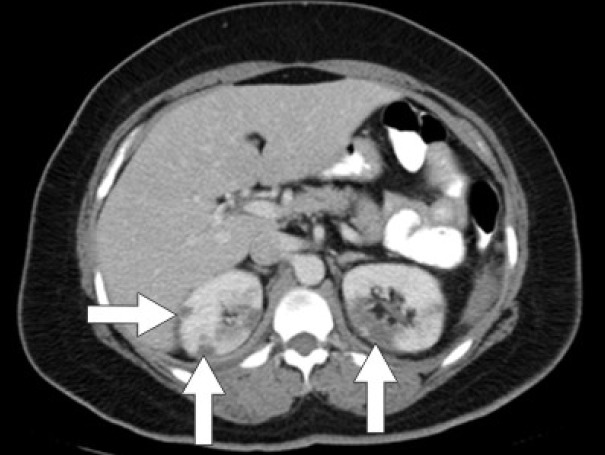
CT scan of the abdomen showing wedge-shaped hypodensities in both kidneys bilaterally consistent with renal infarcts.

**Figure 3:**
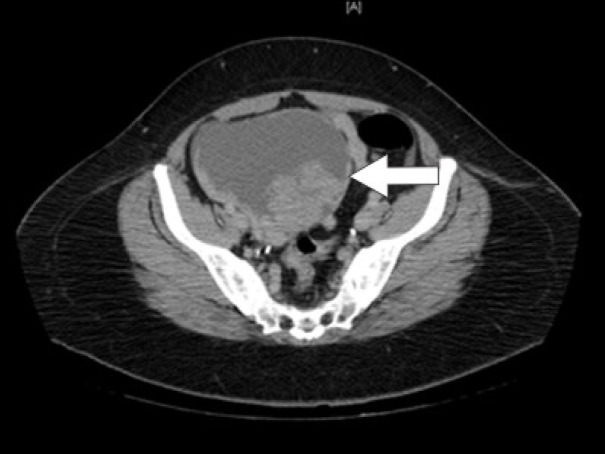
CT scan of the pelvis showing a 12.4 x 8 x 11 cm right pelvic, complex, cystic and solid appearing mass.

**Figure 4:**
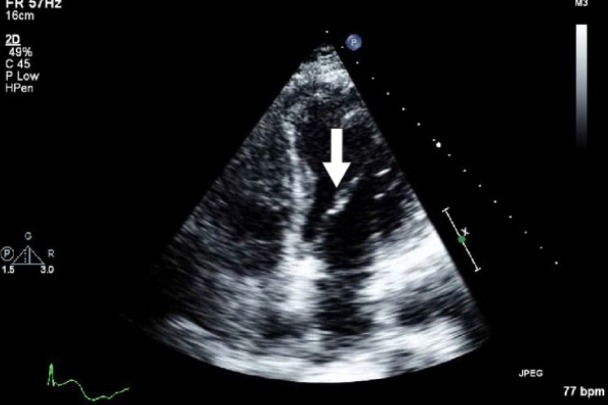
Echocardiogram showing mild mitral regurgitation and a moderate sized vegetation on the anterior mitral valve leaflet.

Initial laboratory studies were as follows: WBC 15.47 x 10/mcL, with a neutrophil count of 11.7 x 10^3^/mcL; hemoglobin 13.3 gm/dL; platelet count 33 x 10^3^/mcL; troponin 4.02 ng/mL; total CK 96 ng/mL, with a CK-MB 2.7 ng/mL and a relative index of 2.5; creatinine 0.8 mg/dL; international normalized ratio (INR) 1.56; PT 18.6 second; PTT 34.6 second; fibrinogen 209 mg/dL; fibrin degradation products (FDP) > 20 mcg/mL, and CA125 of 114 U/mL. Urinalysis showed moderate blood. Her EKG showed findings consistent with an acute, non-ST elevation myocardial infarction. CT imaging showed numerous large splenic, wedge-shaped hypodensities (Fig. 1) with similar areas in the kidneys bilaterally (Fig. 2), most consistent with infarcts. Also noted was a 12.4 x 8 x 11 cm right pelvic, complex, cystic and solid appearing mass (Fig. 3). An echocardiogram was obtained and showed mild mitral regurgitation and a moderate sized vegetation on the anterior mitral valve leaflet (Fig. 4). Lower extremity Duplex showed no apparent thrombosis.

The initial working diagnosis was pelvic malignancy, endocarditis with multi-organ embolic infarction. She was started on appropriate antibiotics; however, over the ensuing days, laboratory studies showed worsening thrombocytopenia, progressive hypofibrinogenemia, and evolving anemia requiring transfusion of platelets, cryoprecipitate and fresh frozen plasma. In addition, she became increasingly weak, with dyspnea, bilateral leg discomfort and edema. A follow-up echocardiogram was unchanged; however, a repeat lower limb venous duplex showed new bilateral major DVT. Serial blood cultures remained negative.

We felt the patient had NBTE related to a pelvic malignancy. She was taken to the operating room for an exploratory laporotomy, total abdominal hysterectomy and bilateral salpingo-oophorectomy. A 12 cm right ovarian cystic mass was completely removed, and pathology confirmed a stage 1C clear cell adenocarcinoma with positive peritoneal fluid cytology. Following surgery, the patient demonstrated steady improvement in her hematologic laboratory studies with normalization of her platelet count and fibrinogen by day 5. She was started on anticoagulation therapy on the third day post-surgery and discharged on warfarin with the intention of completing a 6 months course. A one month follow up echocardiogram showed near complete resolution of the mitral vegetations.

The patient completed 5 cycles of adjuvant chemotherapy and remained well for approximately 2 months, after which she developed extensive bilateral lower extremity DVT with clot up to and beyond the IVC filter. Further imaging showed evidence of a small pulmonary embolism, but no evidence of recurrent malignancy. She was switched to once daily enoxaparin. Several weeks later she was readmitted with acute dysarthria. Brain imaging revealed multifocal, punctuate infarcts, new ascites, evidence of peritoneal carcinomatosis, and further progression of venous thrombosis. No new valvular vegetations were evident by transthoracic echo. Platelet count and coagulation studies remained in the normal range. Cytology obtained from paracentesis confirmed recurrent malignancy. Given the patient’s markedly compromised state and rapid recurrence of malignancy, she was not felt to be a candidate for additional chemotherapy. A few weeks later, the patient died under the care of hospice services.

## Discussion

In 1868 Armand Trousseau described an association between cancer and thrombosis [1]. It is now well established that patients with cancer are at an increased risk of developing and dying from thrombosis. An estimated 15% of patients with cancer will have a thromboembolic event during their lifetime with many more found at autopsy [2, 3]. Although venous thromboembolism is most common, the prothrombotic state induced by malignancy encompasses asymptomatic abnormal coagulation tests, arterial thrombosis, disseminated intravascular coagulation (DIC), and NBTE [4, 5].

NBTE was first described by Ziegler in 1888 and is characterized by valvular thrombus formation, with morbidity and mortality resulting from thromboembolization and organ infarction. Since its original description, several post-mortum case series have identified characteristic features of NBTE, with a reported incidence of 0.3 - 9.3% [6]. It occurs with equal frequency in both genders, from the fourth through the eighth decades of life [6]. Vegetations are composed of sterile platelet and fibrin-based thrombi [7, 8], and primarily involve the mitral valve (64% of cases), followed by the aortic valve (24%), and both mitral and aortic valves (9%) [9].

NBTE occurs most commonly in patients with cancer. In one of the largest autopsy studies, malignancy was present in 51.6% of 217 cases of NBTE [10]. In another large series, a higher prevalence of 78% was observed [11]. Among cancer patients, adenocarcinoma is by far the most common histological subtype. Some case series have also reported an association with hematologic malignancies, particularly leukemia [12, 13].

The pathogenesis of NBTE is incompletely understood but invariably related to the same mechanisms underlying cancer hypercoaguability, notably tumor release of tissue factor and other tumor procoagulants as well as proinflammatory cytokines that indirectly target endothelial cells, leukocytes and platelets. Physical and possibly cytokine mediated valvular injury are believed to contribute to the pathogenesis, accounting for the preferential occurrence of vegetations at sites of high flow [14].

Patients with NBTE may remain asymptomatic or manifest with a clinical embolic event. Valvular vegetations are particularly friable and prone to fragmentation and embolization, often involving multiple organ sites simultaneously, as in our patient. The cerebrovascular bed is the most common site of embolization, with an incidence ranging between 14 and 91% [6]. Other common sites of embolization include coronary, splenic and renal circulations [15-17]. Embolic stroke and myocardial infarction are the major causes of mortality. In addition, DVT and DIC are commonly associated with NBTE. In autopsy studies of patients with malignancy and NBTE, venous thrombosis was present in 26.6% [18], and DIC in 41.9% [10].

NBTE should be suspected in any patient with cancer developing new stroke or other embolic manifestations, with or without venous thrombosis and DIC. The diagnosis is established by echocardiographic evidence of vegetations and negative serial blood cultures. As our case clearly illustrates, the diagnosis of NBTE can antedate the diagnosis of cancer and herald cancer recurrence.

Data regarding the association between NBTE and ovarian cancer is conflicting. Many autopsy reviews failed to identify any patients with NBTE and the diagnosis of ovarian cancer [6]. Two series with the largest prevalence of ovarian cancer include Bedikan [12] (5 patients out of 31) and Kooiker [19] (3 out of 18). Because these autopsy series do not specify ovarian histology, the prevalence of OCCC in NBTE is not known, but likely very low. As far as we know, there is only one published report of NBTE occurring in the setting of OCCC [20]. There is no clear pathogenic mechanism linking OCCC and NBTE; however patients with OCCC appear to have a 2.5 fold greater risk of venous thrombosis compared to those with other histologies [21], raising the question of a clear cell histology driven hypercoagulable state in some patients.

OCCC is an uncommon histological entity, representing approximately 6% of epithelial ovarian tumors [22]. Data regarding prognosis is conflicting; however, when controlled for stage, OCCC tends to be associated with a significantly poorer survival when compared to the more common papillary serous subtype [22]. This was evident in our patient who died of rapidly recurrent and chemotherapy resistant disease, despite a stage-predicted favorable anticipated survival. Although it might be tempting to conclude NBTE is more likely to develop in individuals with an aggressive/resistant tumor phenotype, this association has not been established in other reports.

Our case illustrates how control of the underlying malignancy can lead to prompt resolution of NBTE-associated hypercoaguability. In addition, as has been described by others [23, 24], vitamin K antagonist therapy proved ineffective in controlling thrombosis in our patient. Although heparin and its derivatives have been purported to be more effective than vitamin K antagonist therapy [23, 24], in our patient once a day enoxaparin (1.5 mg/kg) did not prevent progressive thrombosis or thromboembolism from recurrent NBTE (the presumed etiology of her stroke). It remains speculative whether twice daily enoxaparin would have provided better prophylaxis.

Although hypercoaguability is common in patients with cancer, it only rarely manifests as NBTE. Early recognition is difficult but it should be considered in any cancer patient with sudden onset of stroke, myocardial infarction or other arterial thrombosis. As this case illustrates, NBTE can occur in association with a variety of malignancies, including less typical histologic variants, such as OCCC. NBTE may be the initial manifestation of an otherwise asymptomatic, early stage malignancy; furthermore, a return of the prothrombotic state can be a first indicator of disease recurrence. Therapy, when possible, should simultaneously target the underlying malignancy and the malignancy-induced hypercoagulable state. Unfortunately, many patients with NBTE, such as ours, will develop advanced disease with poor outcomes despite aggressive antitumor and anticoagulant therapy.
